# Supplementary material for: Perturbation of protein homeostasis brings plastids at the crossroad between repair and dismantling
Source: PLoS Genet. 2023 Jul 7;19(7):e1010344. doi: 10.1371/journal.pgen.1010344 (PMC10355426; doi:10.1371/journal.pgen.1010344)
Supplement: Attachment — Submitted filename: Rebuttal_Letter.pdf [file pgen.1010344.s024.pdf]

Please find below our point-by-point answers to the major and minor concerns raised by the two reviewers:

Reviewer #2

1. The authors showed that the over-expression of PRPS1 plant show slightly pale true levels, but the authors use the indPRPS1 and indPRPL4 plants infiltrated in presence of DEX for 6 hours in the transcriptome experiment and proteome experiment. I don't know the reason for this? The authors should show the phenotype of the indPRPS1 plant infiltrated in presence of DEX for 6 hours. In the proteome analysis, chloroplast-encode proteins were not changed. However, in the Fig4B, when the indPRPS1 plants are infiltrated in presence of DEX for 6 hours, the amount of PRPS1 protein was significantly reduced. At this point, was chloroplast not impaired? Or could the author explain this?

Reply: The analyses of PRPS1 and PRPL4 over-expressor lines were performed with two different experimental setups in order to reach two different specific goals, aimed to investigate i) long-term and ii) short-term response to the over-accumulation of PRPS1 and PRPL4 transcripts. The long-term effects of PRPS1 and PRPL4 over-expression (Fig. 2 and Fig. S1) differ greatly, indeed. While PRPS1 over-expression leads to altered chloroplast development, the activation of related degradation pathways (Fig. 3 and Fig. S2), and early flowering (Fig. 9), PRPL4 over-expression does not influence chloroplasts integrity and functionality (Fig. 2 and Fig. 3). To dissect the early cellular events that, later on, result into chloroplast degradation in PRPS1-oeverexpressor lines, the short-term PRPS1 and PRPL4 induction was studied within 24-hour timeframe using different approaches. The results show that PRPS1 over-expression fails to accumulate more PRPS1 protein. At 6 hours from induction, PRPS1 transcripts reached a peak of accumulation but PRPS1 protein level was decreased, together with other ribosomal proteins (Fig4 A and B). These observations suggest that at 6 hours the short-term effects are detectable, as plastid ribosome and translation capability are affected (Fig.4 B, D and E). Nevertheless, this time point was also selected since no significant differences arose in terms of visible phenotype and photosynthetic efficiency upon 6 hours of incubation with DEX with respect to the mock control (-DEX), as shown in the new Fig. S9, reducing at minimum the consequences of pleiotropic effects and allowing to detect at the very beginning the specific consequences of the alteration of plastid proteostasis.

2. The transcript levels of chloroplast-encode genes were not altered in the transcriptome. The transcriptome analysis methods should be described in more detail. Especially, how to build the libraries. This is related to the quantification of chloroplast gene.

Reply: We have clarified in the Materials and Methods section, paragraph "Transcriptome analysis" that the RNA-seq library was prepared by enriching for PolyA-containg transcripts, therefore no

plastid-encoded genes are represented in the library and their differential expression cannot be inferred from our transcriptome data.

3. PRPS1 as a member of plastid ribosome, the amount of PRPS1 protein was significantly reduced when the indPRPS1 plants infiltrated in presence of DEX for 6 hours. I think the chloroplast may not be under the normal condition, the transcription level and translation level still remained unchanged? And the author also investigated the possible negative effect of PRPS1 inducible expression on plastid protein translation. In this paper, I think the transcription level and protein level of chloroplast-encode genes should be shown because the authors aimed to describe the effect of perturbance chloroplast protein homeostasis.

Reply: The RNA-seq library was prepared enriching the PolyA-containing transcripts only, therefore, no plastid-encoded genes are represented. The way the library was generated is now detailed in the Materials and methods section. To evaluate if plastid transcription was affected by the DEX-mediated induction the very same samples used for transcriptome analyses were analyzed via RT-qPCR to quantify the expression of a subset of PEP- and NEP-dependent plastid-encoded genes (see new Fig. S12), on the very same samples employed for the transcriptome analysis. The results showed no significant differences in the expression of these genes in both lines after DEX treatment. On the other hand, the translation rate of indPRPS1 lines supplemented with DEX showed great impairment of plastid-encoded protein synthesis (Fig. 4D). Furthermore, an alteration of chloroplast-related proteins emerges from the proteomic investigation. As reported (see Tables S7, S8 and the corresponding text in the Results section), among over-represented GO terms in down-accumulated proteins we found "chloroplast stroma" (GO:0009570) and "chloroplast organization" (GO:0009658). In the latter category, we observed reduced amounts of proteins, such as the chloroplast-localized nifu-like protein 2 (AT5G49940), the plastid transcriptionally active 15 (mTERF8; AT5G54180) and a close homolog of the Cauliflower OR (AT5G61670), which suggest a possible impairment of chloroplast functions.

4. In the Fig4D, the contents of RbcL and D1/D2 showed a big difference in vivo. The blots may be over exposed in order to quantitative analysis. The author can check the two proteins in different blot.

Reply: The figure (Fig. 4D) does not display a western blot, but an in vivo protein synthesis rate assay. It displays the protein synthesis rate of the plastid-encoded genes over time and gives an idea of the plastid translation capability. After incubation with radio-labelled ^{35}S-methionine and light exposure, samples were collected at 15 min and 30 min. The experimental procedure exploits cycloheximide, that blocks the cytosolic translation thus allowing the synthesis of the plastid-encoded proteins only. RbcL and D1/D2 proteins results in the most prominent signals, just because the synthesis rate is the highest among plastid-encoded proteins (Romani et al., 2012).

5. The author think PRPS1 accumulation is negatively regulated by chloroplast Clp protease complex. The accumulation of PRPS1 protein increased about two-fold in all the double mutants tested with respect to prps1-1. In the Fig6C, the CBB stain is not precise in my eyes. Thought I believe the results, the authors should check the results of protein quantification by examining gene Actin.

Reply: To address this point we used the nuclear-located histone H3 as loading control, since it appears to be a better control than Actin in mutants with a marked leaf phenotype, where defects in cell cytoskeleton cannot be excluded (see new Fig. 6).

6. As for the overexpression PRPS1 material, I am also curious the protein level of PRPS1 was dramatically decreased. This point should be clarified. One point is that if all PRPPS1 proteins can enter the chloroplast, or part of them stay in the cytoplasm and then be degraded. Or is it likely the transgenetic plants carry a second mutation?

Reply: To address this point and to exclude the side-effects of T-DNA insertion itself, data from independent over-expressor and inducible PRPS1 lines have been provided (see new Fig. S1). Moreover, in oePRPS1 lines, PRPS1 protein accumulates in the chloroplast fraction only (Fig. S8), while there is no trace of it in extra-plastid fractions, suggesting that PRPS1 is imported in the plastid before degradation occurs. To strengthen both these points, we crossed oePRPS1 with clpc1-1 and clpd-1 mutants, lacking two subunits of CLP plastid-located protease complex, showing that the accumulation of PRPS1 protein is partially recovered, pointing to a major role CLP in plastid degradation of PRPS1 protein (Fig. S7). Similarly, the increased accumulation of PRPS1 protein could be observed upon short-term induction of PRPS1 in clpc1-1 and clpd-1 mutant backgrounds, when compared to indPRPS1 +DEX samples (Fig. 6 D and Fig. S7 A). Finally, no peptides from PRPS1 cTP could be detected in our proteomics studies. Overall, this set of evidences point to a complete import of PRPS1 protein into the chloroplast stroma, where its abundance is modulated by the CLP protease complex, as part of the the plastid proteostasis machinery.

Reviewer #3

Major points:

1. The lack of genetic material makes the genetic data of this manuscript unconvincing. The transgenic plants (oePRSP1, indPRPS1, oePRPL4 and indPRPL4) used in this paper have only one line respectively. Please provide other independently transformed transgenic lines and their corresponding data, including the phenotypes and the expression levels of relevant RNA and protein.

Reply: To address this point, data from three independent over-expressor and inducible lines for each genotype have been added. Visible phenotypes, photosynthetic efficiency, expression of PRPS1 and PRPL4 at transcript and protein level are now shown in the new Fig. S1. Moreover, several oePRPS1

lines have been already described in literature (Yu et al. 2012 and Tadini et al. 2016). The text referring to the new Fig S1 has been added in the paragraph "PRPS1 over-expression impairs chloroplast activity and biogenesis" of the Result section and in the legend of Fig. 2. Additionally, a new Fig. S1 and the corresponding legend has been added to the supplementary figure section.

2. The authors concluded that "the accumulation of PRPS1 is negatively regulated by chloroplast CLP protease complex" by comparing the accumulation of PRPS1 in the prps1-1 mutant and prps1-1 clp double mutants. prps1-1 is a knockdown mutant of PRPS1, and the increased amount of PRPS1 protein in prps1-1 clp double mutants is not sufficient to draw this conclusion. Why is the accumulation of PRPS1 protein not higher in clp mutants than in wild-type plants? This problem can be better explained if indPRPS1 is crossed with the clp mutants.

Reply: To address this concern, two different indPRPS1 transgenic lines have been crossed with clpc1-1 and clpd-1 mutant backgrounds and the accumulation of PRPS1 protein was tested upon DEX induction (0-24 h) (see Fig. 6 and Fig. S7). Compared to indPRPS1 lines, in which PRPS1 accumulation decreases over time, PRPS1 accumulation level remained rather stable upon induction in plastids with defects in the CLP-mediated protein degradation. Similarly, oePRPS1 clpc1-1 and oePRPS1 clpd-1 lines showed higher PRPS1 accumulation and partial rescue of the photosynthetic phenotype when compared with oePRPS1 plants (Fig. S7), corroborating the notion that the CLP protease is responsible for PRPS1 degradation.

3. To investigate the molecular responses to the increased expression of PRPS1 gene, transcriptome and proteome analysis was performed on leaf discs harvested from indPRPS1 that were vacuum infiltrated for 6 hours in either the absence or presence of DEX. As shown in Figure 4, after 6 hours DEX induction, the RNA expression level of PRPS1 increased 50-fold, while the protein level of PRPS1 decreased to 40%. Therefore, it is difficult to tell whether the transcriptomic and proteomic results are caused by increased PRPS1 gene expression or decreased PRPS1 protein content. Figure 2 showed that compared to WT, the expression level of PRPS1 gene is 20% and the protein content is 45% in prps1-1 mutant. If the prps1-1 mutant is added as a reference in transcriptome and proteome analysis, it will be helpful to draw the correct conclusion.

Reply: While we agree with R#3 that this point needs a clarification, we do not think that the strategy he/she suggested provide the desired information. Indeed, the comparative analysis of indPRPS1, where the transient over-expression PRPS1 is triggered, with prps1-stable lines, in which PRPS1 expression is reduced throughout the entire plant life cycle, represent two completely different experimental setups difficult to be compared. However, to clarify the reviewer's concerns, we have analyzed the accumulation of TULP5 and SWEET13 (see new Fig. S14) transcripts (highly up-regulated in indPRPS1 in response to DEX) by comparing their expression indPRPS1 + DEX, in which the up-regulation of PRPS1 transcript is followed by PRPS1 degradation, to indPRPS1 clpd-1 + DEX, in which PRPS1 degradation is inhibited, despite similar PRPS1 transcript upregulation (Fig 6 and S7). Interestingly, TULP5 and SWEET13 transcripts did not over-accumulate in the

absence of a functional CLP protease, indicating that the re-orchestration of nuclear gene expression requires the accumulation/degradation of PRPS1 protein (Fig. S14). Similarly, transgenic lines in which the prps1-2 null allele is either under the control of CaMV35S promoter or the inducible promoter (see Materials and Methods) in Col-0 wild-type background did not show any virescent phenotype, supporting the notion that, in order to trigger its own degradation, the transient over-accumulation of PRPS1 protein is required (Fig. S14).

Minor points:

4. In Figure 2, the alteration of PRPS1 expression has different effects on true leaves and cotyledons. Please explain it.

Reply: Chloroplasts in cotyledons and true leaves were often found to behave differently in mutants lacking factors involved in plastid biogenesis, protein homeostasis and degradation (Chen et al. 2000; Albrecht et al. 2006; Jeran et al. 2020; Tadini et al. 2020). Thus, it is possible that alterations in PRPS1 homeostasis has different impacts on the two organs. This aspect has been highlighted in the discussion section.

5. Please add information about prps1-1 mutants.

Reply: Information related to the Arabidopsis prps1-1 mutant was already included in the Materials and Methods section and in the Introduction section together with the corresponding reference (Romani et al. 2012). A further piece of information related to the position of T-DNA insertion responsible for the prps1-1 knock-down allele has been added in the Introduction section.

6. There are three pathways of chloroplast degradation: senescence-associated vacuoles (SAVs), chloroplast vesiculation, and autophagy. In order to accurately analyze chloroplast degradation in Figure 3, marker lines of different degradation pathways should be added as controls, or marker genes of different degradation pathways should be comprehensively examined.

Reply: The expression level of several genes involved in different vacuole-mediated chloroplast degradation pathways was already reported in the former Fig. S1 that in the current version of the manuscript is indicated as Fig. S2. We believed to have provided a comprehensive picture of the chloroplast degradation mechanisms by including genes involved in ATG-dependent and -independent pathways. However, the SAV pathway (Izuma and Nakamura, 2018) that was not considered in the first version of the manuscript, was now investigated by monitoring the expression

of SAG12 gene. However, the SAG12 transcripts were below the limit of detection, probably because the SAV pathway is mainly activated during senescence rather than upon stress conditions.

7. Please explain the calculation method of Figure 4F.

Reply: the following sentence has been added to the paragraph "Isolation of PRPS1-containing protein complexes" in the Materials and Methods section: "Quantification of PRPS1 protein accumulation in Low Molecular Weight (LMW; fractions 1-5) and High Molecular Weight (HMW, fractions 6-9) fractions was evaluated by using the Image Lab software on representative blots obtained from three biological and nine technical replicates (three technical replicates for each biological replicate). Each lane has been quantified as absolute value. The sum of all signals has been set as 1. The values reported in the graph are relative to the total sum for each condition (DEX -/+).
